# Overcoming Tumor Resistance to Oncolyticvaccinia Virus with Anti-PD-1-Based Combination Therapy by Inducing Antitumor Immunity in the Tumor Microenvironment

**DOI:** 10.3390/vaccines8020321

**Published:** 2020-06-19

**Authors:** So Young Yoo, Narayanasamy Badrinath, Su-Nam Jeong, Hyun Young Woo, Jeong Heo

**Affiliations:** 1 BIO-IT Foundry Technology Institute, Pusan National University, Busan 46241, Korea; sukhan@nate.com; 2 Research Institute for Convergence of Biomedical Science and Technology, Pusan National University Yangsan Hospital, Yangsan 50612, Korea; badrisamy@gmail.com; 3 Biomedical Sciences, School of Medicine, Pusan National University, Yangsan 50612, Korea; 4 Department of Internal Medicine, College of Medicine, Pusan National University and Medical Research Institute, Pusan National University Hospital, Busan 49241, Korea; who54@hanmail.net

**Keywords:** oncolytic virotherapy, immunotherapy, tumor microenvironment, vaccinia virus, immune checkpoints

## Abstract

The tumor microenvironment (TME) comprises different types of immune cells, which limit the therapeutic efficacy of most drugs. Although oncolytic virotherapy (OVT) boosts antitumor immunity via enhanced infiltration of tumor-infiltrated lymphocytes (TILs), immune checkpoints on the surface of tumors and TILs protect tumor cells from TIL recognition and apoptosis. OVT and immune checkpoint blockade (ICB)-based combination therapy might overcome this issue. Therefore, combination immunotherapies to modify the immunosuppressive nature of TME and block immune checkpoints of immune cells and tumors are considered. In this study, cancer-favoring oncolytic vaccinia virus (CVV) and anti–programmed cell death protein-1 (anti-PD-1) were used to treat mouse colorectal cancer. Weekly-based intratumoral CVV and intraperitoneal anti-PD-1 injections were performed on Balb/c mice with subcutaneous CT26 tumors. Tumor volume, survival curve, and immunohistochemistry-based analysis demonstrated the benefit of co-treatment, especially simultaneous treatment with CVV and anti-PD-1. Infiltration of CD8^+^PD-1^+^ T-cells showed correlation with these results. Splenocytes enumeration also suggested CD4^+^ and CD8^+^ T-cell upregulation. In addition, upregulated CD8, PD-1, and CD86 messenger RNA expression was observed in this combination therapy. Therefore, CVV+anti-PD-1 combination therapy induces antitumor immunity in the TME, overcoming the rigidity and resistance of the TME in refractory cancers.

## 1. Introduction

Oncolytic virotherapy (OVT) uses oncolytic viruses (OVs) to treat cancer. OVs have the intrinsic ability or are genetically modified to selectively infect tumor cells [1]. OVs can induce both local and systemic immunity against tumor cells. Oncolysis induces immunogenic cell apoptosis, pathogen-associated molecular patterns, danger-associated molecular pattern signals, and tumor-associated antigens (TAAs) [2]. To induce robust tumor cell apoptosis, various immune modulatory transgenes are inserted into OVs, and preclinical and clinical studies have documented the therapeutic efficacy of these OVs [3,4,5,6]. Although OVT shows promising results, its therapeutic efficacy is limited by immune cells in the tumor microenvironment (TME) [7].

Tumor cells induce infiltration of various immune cells and form a TME [8]. Immune cells in the TME produce various cytokines, most of which promote tumor formation and decrease intrinsic antitumor immunity. The TME comprises tumor-associated macrophages (TAMs), myeloid-derived suppressor cells, tumor-associated neutrophils, and terminally differentiated myeloid dendritic cells [9]. All these are myeloid lineage immune cells, abundantly found in the TME, and severely affect the therapeutic efficacy of various cancer therapies. Stromal cells such as cancer-associated fibroblasts, vascular endothelial cells, pericytes, and adipocytes also infiltrate the TME, and these cells are also involved in tumor progression and severely affect therapeutic efficacy [10].

The immune checkpoint axis comprises receptors and their corresponding ligands to recognize self- and non-self-antigens [11]. Tumor cells express programmed cell death ligand-1 (PD-L1) and programmed cell death ligand-2 (PD-L2) on their membranes. Activated T-cells express programmed cell death protein-1 (PD-1), which is a receptor for PD-L1 and PD-L2. Cytotoxic T-lymphocyte-associated antigen-4 (CTLA-4) is also expressed on lymphocytes. Receptor and ligand binding of immune checkpoints inhibits T-cell recognition and tumor antigen presentation [12]. Current therapeutic strategies, especially immunotherapies such as OVT, immune checkpoint blockade (ICB), and chimeric antigen receptor T-cells, enhance lymphocyte infiltration and activation in the TME [13]. Although each therapy has potential therapeutic efficacy, it is limited because of the TME’s immunosuppressive nature [14]. Other cells have also reportedly infiltrated the TME; however, these cells and the immune checkpoint axis decrease tumor cell recognition by lymphocytes, which leads to innate and adaptive immune resistance [15]. In this case, combination therapy would be an ideal option for inducing antitumor immunity in the TME.

Vaccinia virus (VV) belongs to poxviridae family, and consists of 190kb double DNA as a genome. Due to its large genome size, and good tolerance in humans, VV has been widely engineered as a vector in OVT. For vector construction, three major strains of VVs such as Wyeth, Western Reserve and Lister strains have been used [1]. Disruption in viral thymidine kinase (vTK) and deletion of vaccinia virus growth factor (VGF) are used to selectively infect and replicate in cancer cells [16]. In order to induce anticancer immunity, various cytokines can be inserted into VV, such as therapeutic genes, and their therapeutic efficacy was evaluated in preclinical and clinical studies [3,5,6,17]. The advantages of using VV include its utility as a cancer vaccine because of its high immunogenicity [18,19]. To support the immunostimulatory effect of VV, combining virus administration with ICB can be an optimal option for improving anti-tumor immunity. Recent studies have shown considerable therapeutic efficacy of OVs in combination with ICB in various cancer models [20,21]. However, time interval, dosage, and toxicity play a crucial role in combination therapy [22]. OV and ICB combination therapy needs more attention because both agents activate the immune system, which might lead to systemic immune adverse effects.

In this study, we used cancer-favoring oncolytic vaccinia virus (CVV) and anti-PD-1 to treat mouse colorectal cancer (CRC). CVV was demonstrated previously in our group; the released virus into the serum of a Wyeth strain vaccinia virus-injected VX2 tumor animal model during the tumor volume reduction was isolated and subsequently engineered by deleting the Tk gene to get the virus with enhanced cancer selectivity (called CVV) [23]. Without any therapeutic transgene inserted into CVV, the therapeutic efficacy of CVV was evaluated in colon cancer [23,24], cholangiocarcinoma [25] and hepatocellular carcinoma models [26]. Herein, we exploited how CVV and anti-PD-1 combination therapy induces the antitumor immunity in the TME to overcome the rigidity and resistance of the TME in refractory cancers.

## 2. Materials and Methods

### 2.1. Cell Culture

Cancer cell lines were purchased from Korean cell line bank (South Korea). PANC-1, MIA PaCa-2, and Sk-Hep-1 cell lines were cultured in Dulbecco’s Modified Eagle Medium (DMEM) containing 10% heat-inactivated fetal bovine serum (FBS) and 1% penicillin/streptomycin (P/S). The other cell lines, SNU354, HepG2, CT26, HT-29, LoVo, HuCCT-1, SNU-1196 and Capan-1 were cultured in Roswell Park Memorial Institute (RPMI) 1640 medium containing 10% FBS and 1%P/S. Cell culture media and reagents were obtained from Welgene (Daegu, South Korea). Cell were grown in 5% CO_2_ and 37 °C condition in CO_2_ incubator.

### 2.2. CVV Amplification and Purification

The CVV was generated through evolution of the Wyeth strain of the VV, deletion of thymidine kinase, and insertion of the green fluorescence protein (GFP) and guanine-hypoxanthine phosphoribosyltransferase (GPT) selection markers. The human cervical cancer cell lines HeLa (ATCC) were infected with CVV in Dulbecco’s modified Eagle medium (DMEM; Welgene) supplemented with 2% fetal bovine serum (FBS; Welgene) for 2 h. Then, medium was replaced with GPT selection media (DMEM with 10% FBS, 25 µg/mL Mycophenolic Acid, 250 µg/mL Xanthine, and 15 µg/mL Hypoxanthine) and infected cells were cultured in selection media for 72 h. The cells and medium were then harvested and homogenized. The amplified virus in homogenized samples were purified through sucrose cushion centrifugation consisting of 36% sucrose in 10 mM Tris, pH 9.0, and purified virus was re-suspended in the final stock buffer, 1 mM Tris, pH 9.0.

### 2.3. WST-1 Assay

**Figure 1:**
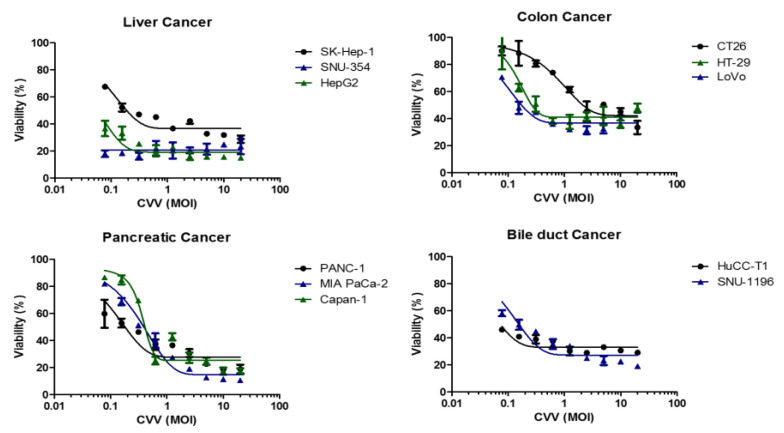
Oncolytic activity of the cancer-favoring oncolytic vaccinia virus (CVV) in different type of cancer cell lines. The viability (%) of four different cancer types (liver, colon, pancreas, bile duct) against the CVV at different multiplicity of infection (MOI).

For the in vitro cytotoxicity assay, 10,000 cells were seeded in 96 well plates. After 24 h, CVV was infected dose-dependent manner (0.7~20 multiplicity of infection (MOI)) with corresponding serum free media (Figure 1). This setup was maintained for 2 h for virus inoculation, and then corresponding media with serum were replaced. After 72 h of infection, media were removed from the wells, 100 µL of 10% WST-1 reagent was added to assess cellular cytotoxicity. After 2 h, optical intensity was quantified in 450 nm (PerkinElmer, Waltham, MA, USA).

### 2.4. Animal Experiments

**Figure 2:**
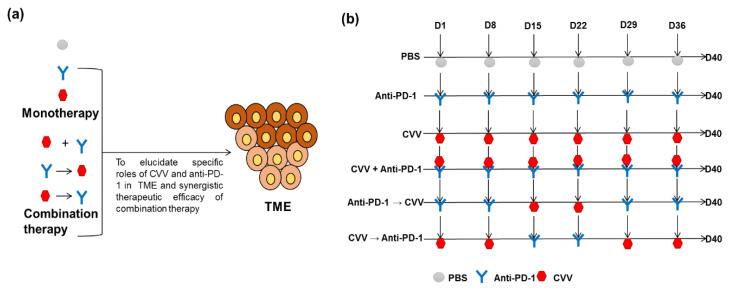
Monotherapy and combination therapy of CVV and anti–programmed cell death protein-1 (anti-PD-1). (**a**) Combination therapy strategies. To reveal the specific effects of CVV and anti-PD-1 in the therapeutic efficacy of combination therapy, mice were divided into three groups: CVV+anti-PD-1, anti-PD-1→CVV, and CVV→anti-PD-1. (**b**) Weekly combination therapy schedule. Subcutaneous CT26 tumors were induced in six-week-old male Balb/c mice using 5 × 10^6^ CT26 cells/100 µL of 1X phosphate-buffered saline (PBS). After the tumor volume reached 100 mm^3^, mice were separated into different groups for treatment (*n* = 8–10 in each group). The control group received 100 µL of 1X PBS up to six weeks (D1, D8, D15, D22, D29, D36, and D40). The anti-PD-1 group received 250 µg of RMP1-14 intraperitoneally up to six weeks. The CVV group received 1 × 10^7^pfu of CVV intratumorally up to six weeks. The CVV+anti-PD-1 group received both RMP1-14 and CVV on the same days up to six weeks. The anti-PD-1→CVV group received RMP1-14 consecutively for the first two weeks (D1 and D8), followed by CVV for the next two weeks (D15 and D22) and RMP1-14 injection was repeated for the last two weeks (D36 and 40). The CVV→anti-PD-1 group received CVV consecutively for the first two weeks (D1 and D8), followed by RMP1-14 for the next two weeks (D15 and D22), and CVV injection was repeated for the last two weeks (D36 and 40). The dosage and route of administration were the same as in the monotherapy groups. Two independent experiments were carried out as per the above schedule.

This study was approved by the institutional animal care and use committee of Pusan National University, South Korea (the ethical code: PNU-2019-2391). We purchased 6-week-old male Balb/c mice from Orient (South Korea) and kept them in pathogen-free conditions in animal facilities of the medical school of Pusan National University. To evaluate the therapeutic efficacy of CVV and anti-PD-1 alone or in combination (Figure 2a), we designed a study protocol with six groups (Figure 2b). We scheduled weekly treatment, considering the possible toxicity of anti-PD-1 and CVV-induced immunogenic cell apoptosis. We also tried to determine the effects of CVV and anti-PD-1 on the TME. CT26 tumors were induced on the left flank of the mice using 5 × 10^6^ CT26 cells/100 µL of 1X phosphate-buffered saline (PBS). Tumor volume was measured twice weekly using a caliper in two dimensions, and tumor burden was calculated as tumor volume (mm^3^) = (length × width2)/2. Treatment was begun once the tumor volume reached >100 mm^3^. Weekly intratumoral injections of 1 × 10^7^ plaque-forming units (pfu) of CVV in 100 µL of 1X PBS and intraperitoneal injections of 250 µg of anti-PD-1 (RMP1-14, PharmaEssentia Corp., Taipei, Taiwan) in 100 µL of 1X PBS were scheduled to avoid toxicity (days 1, 8, 15, 22, 29, and 36). Mice given PBS were treated as the control group. The two monotherapy groups were administered either CVV or anti-PD-1. In addition, to assess the synergistic efficacy of combination therapy, the mice were divided into three more groups: CVV+anti-PD-1, anti-PD-1→CVV (alternative anti-PD-1 and CVV administration), and CVV→anti-PD-1 (alternative CVV and anti-PD-1 administration) (Figure 1). At day 40, mice were euthanized by CO_2_ inhalation in a chamber. Two independent experiments were carried out as per the above schedule.

### 2.5. Flow Cytometry and Immunophenotyping of Splenocytes

The following antibodies were used for staining cell surface markers: CD45-PE (#B251876, Biolegend, San Diego, CA, USA), CD8-PE (#130-109-247, MiltenyiBiotec GmbH, BergischGladbach, Germany), and CD4-PE (#130-116-509, MiltenyiBiotec GmbH). The mice were euthanized and spleens were collected for flow cytometry. Briefly, the whole spleen was treated with RBC lysis buffer and smashed thoroughly between frosted glass slides until it dissociated. Then it was transferred to a tube containing 10 mL of RPMI 1640 medium (10% FBS, 1% P/S, and 5 mM β-mercapto-ethanol) and inverted three to four times. To isolate splenocytes, this solution was centrifuged at 1300 rpm for 5 min at 4 °C. The pellet was filtered through a 70 μM filter over a 50 mL conical tube. A Navios flow cytometer (Beckman Coulter, Indianapolis, IN, USA) was used for immunophenotyping of splenocytes. The splenocytes were counted and stained with respective antibodies for immune cell counting according to the manufacturer’s instructions.

### 2.6. Hematoxylin and Eosin Staining, Immunohistochemistry, and Immunofluorescence Staining

Paraffin-embedded tumor sections were used for hematoxylin and eosin (H&E) staining, IHC, and immunofluorescence staining. For immunofluorescence staining, sample fixation was done using 4% paraformaldehyde. Sample permeabilizationwas done using 0.1% Triton X-100, and 5% normal goat serum was used to block nonspecific binding. Unlabeled primary anti-CD8 (Abcam, Cambridge, UK) and anti-PD-1 (Bioxcell, Lebanon, NH, USA) antibodies and fluorophore-conjugated secondary antibodies were used for staining. Stained samples were examined under a fluorescence microscope (Nikon Eclipse Ni, Melville, NY, USA). ImageJ 1.52a software (National Institutes of Health, Bethesda, MD, USA) was used to quantify reciprocal and fluorescence intensities of the samples.

### 2.7. Real-Time Polymerase Chain Reaction

Total RNA was extracted from harvested tumors using Trizolreagent (Life Technologies, Carlsbad, CA, USA) in accordance with the manufacturer’s instructionsand quantified using Nano Drop (both from Thermo Fisher Scientific, Wilmington, DE, USA). cDNA was synthesized from the extracted RNA using an RT Synthesis kit (INTRON Biotechnology, Seongnam, South Korea). A total of 2 µg of cDNA was mixed with SYBR Green I master mix (Roche, Basel, Switzerland) and respective primers (Table 1). The Light Cycler 96 Real-Time PCR System (Roche) was used to perform qPCR. The qPCR program consisted of 45 cycles of amplification for 10 s at 95 °C, for 10 s at 60 °C, and, finally, for 10 s at 72 °C. β-actin was used as reference gene to normalize the relative expression of genes, and the expression was quantified using the ΔΔCt method: ΔΔCt = mean(ΔCt_treated_) − mean(ΔCt_control_), where ΔCt_treated_ = Ct_reference gene, treated_ − Ct_target gene, treated_, ΔCt_control_ = Ct_reference gene, control_ − Ct_target gene, control_.

### 2.8. Statistical Analysis

All statistical analyses were performed using GraphPad Prism 5 software (GraphPad Software Inc., San Diego, CA, USA). One-way analysis of variance and Tukey’s multiple-comparison test were used to compare the means of all groups. The log-rank test was used for survival differences in Kaplan–Meier curves. Tumor volume >1000 mm^3^ was censored for survival curve analysis; *p* < 0.05 was considered statistically significant.

## 3. Results

### 3.1. Cytotoxicity of CVV in Various Cancer Cell Lines

To select the refractory cancer model in CVV monotherapy, cytotoxicity of CVV in vitro was evaluated using various cancer cell lines. Sk-Hep-1, SNU354, HepG2 (human liver cancer), CT26 (mouse colon cancer), HT-29, LoVo (human colon cancer), PANC-1, MIA PaCa-2, Capan-1 (human pancreatic cancer) and HuCCT-1, SNU-1196 (human bile duct cancer) were infected with CVV at different MOIs. Although most types of cancer cells respond to CVV with considerable cytotoxicity in CVV-dose dependent manners, mouse colon cancer CT26 shows a somewhat attenuated response to CVV compared to other cell lines (Figure 1). Therein, CT26 was used to generate the colon cancer model for this combination immunotherapy, since we considered combination therapy in a CT26 syngeneic immunocompetent mouse CRC model and focused on dosage and the route of administration, because these two factors play an important role in the therapeutic efficacy of combination therapy.

### 3.2. Monotherapy and Combination Therapy of CVV and Anti-PD-1 and Their Therapeutic Efficacy

To evaluate the therapeutic efficacy of CVV and anti-PD-1 in monotherapy or combination therapy, we designed this study protocol with six groups (PBS control group and other five test groups in Figure 2a). Considering the possible toxicity of anti-PD-1 and immunogenic cell death by CVV, we scheduled weekly-based treatments. Efforts were also made to elucidate impacts of CVV and anti-PD-1 on TME. As a syngeneic immune-competent CRC model, CT26 tumors were induced on the left flank of Balb/c mice. Weekly-based intratumoral injection of CVV (1 × 10^7^ pfu in 100 µL 1X PBS) and/or intraperitoneal injection of anti-PD-1 (250 µg in 100 µL 1X PBS) was scheduled in order to avoid toxicity (day 1, day 8, day 15, day 22, day 29 and day 36). The treatment was started once tumor volume reached >100 mm^3^. Mice in the PBS group received PBS, as the control group. Monotherapy groups received either CVV or anti-PD-1. To assess the synergistic efficacy of combination therapy, three groups were used; CVV and anti-PD-1 were simultaneously injected in the CVV+anti-PD-1 group. Anti-PD-1 was injected at day 1 and day 8, which was followed by CVV injection at day 15 and day 22; at day 29 and day 36, again, anti-PD-1 was injected in Anti-PD-1→CVV group. In the CVV→anti-PD-1 group, CVV was injected at day 1 and day 8 and was followed by anti-PD-1 injection at day 15 and day 22; at day 29 and day 36, again, CVV was injected (Figure 2b).

### 3.3. Responders and Nonresponders According to Tumor Burden

**Figure 3:**
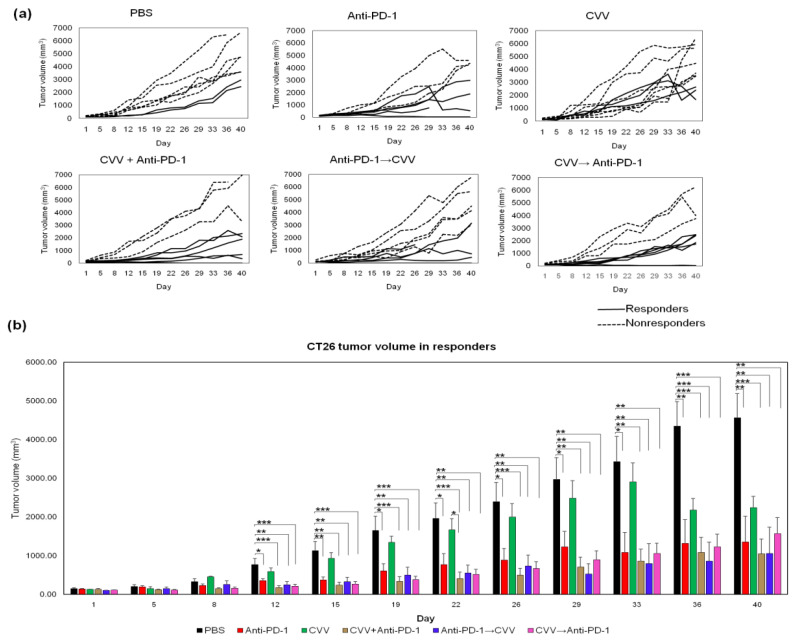
Monotherapy and combination therapy of CVV and anti-PD-1 and their therapeutic efficacy. (**a**) CT26 tumor burden in Balb/c mice after combination therapy. CT26 cells (5 × 10^6^ cells/100 µL of 1X PBS) were used to induce subcutaneous tumors in the left flank of Balb/c mice. Tumor burden was monitored twice weekly. When the tumor volume reached >100 mm^3^, treatment was started (solid lines: responders, dashed lines: nonresponders). (**b**) On the basis of the tumor burden, mice were categorized as responders or nonresponders. Tumor volume in responders. * *p* < 0.05; ** *p* < 0.01; *** *p* < 0.001. CVV, cancer-favoring oncolytic vaccinia virus; PD-1, programmed cell death protein-1; PBS, phosphate-buffered saline; D1, day 1; D8, day 8; D15, day 15; D22, day 22; D29, day 29; D36, day 36; D40, day 40.

The overall tumor burden was recorded twice weekly. Complete tumor regression was observed in a few mice in combination therapy groups: CVV+anti-PD-1, 33.33%; anti-PD-1→CVV, 20%; and CVV→anti-PD-1, 14.28%. We categorized the mice into responders or nonresponders on the basis of each mouse’s tumor response; the control group tumor burden was used to set the cut-off value. In the control group, the average tumor burden increase from days 1 to 40 was ~3000% ((V − V_0_)/V_0_ × 100), therefore we categorized mice with a tumor burden increase <3000% as responders (Figure 3a, solid lines) and others as nonresponders (Figure 3a, dashed lines). Among responders, we observed a significantly slow tumor burden in combination therapy groups, especially the CVV+anti-PD-1 group, compared to the control, CVV, and anti-PD-1 groups from days 12 to 40. (Figure 3b). A slower tumor burden and complete tumor regression in combination therapy groups confirmed the therapeutic efficacy of combination therapy.

### 3.4. Prolonged Survival Due to CVV+Anti-PD-1 Combination Therapy

**Figure 4:**
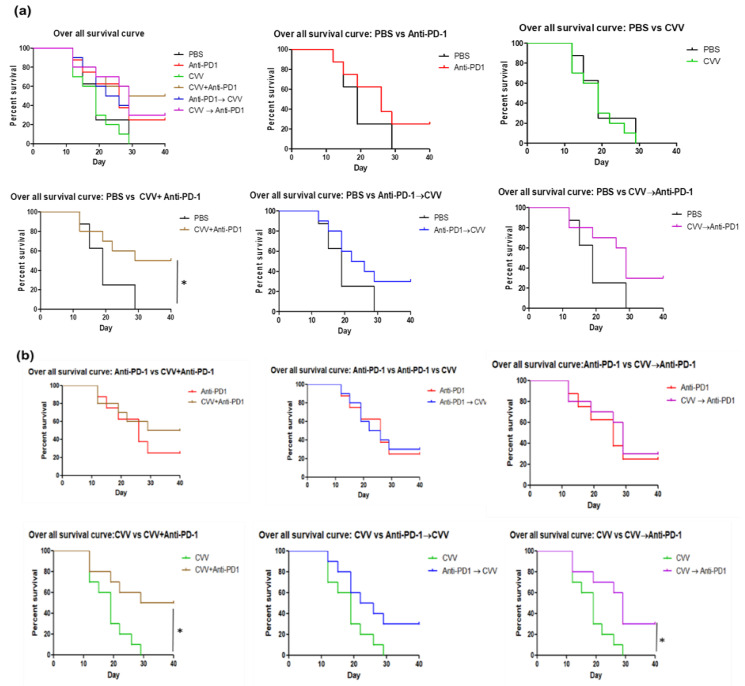
Survival curve analyses. Kaplan–Meier curves were plotted on the basis of tumor volume. Tumor volume >1000 mm^3^ was considered death. (**a**) Overall survival curve. (**b**) Overall survival curve: monotherapy vs. combination therapy. * *p* < 0.05.

In survival curve analysis, a tumor volume of 1000 mm^3^ was considered death. The overall survival curve analysis showed prolonged survival of the CVV+anti-PD-1 group compared to the control group (Figure 4a) and prolonged survival of the CVV+anti-PD-1 and CVV→anti-PD-1 group compared to the CVV group (Figure 4b). As for the adverse effect of the combined therapy, we did not observe any mortal cases with unknown reasons in the combination therapy groups. Among responders, all groups except the CVV group showed significant survival compared to the control group (Figure S1a). In addition, responders showed prolonged survival in CVV+anti-PD-1, anti-PD-1→CVV, and CVV→anti-PD-1 groups compared to the CVV group. There was no significant survival observed between anti-PD-1 monotherapy and dual therapies received groups (Figure S1b). These results indicate the therapeutic efficacy of weekly simultaneous CVV+anti-PD-1 combination therapy in murine colon cancer, especially the enhanced efficacy of OVT.

To elucidate immune cell infiltration, next we examined CD8^+^, PD-1^+^, PD-L1^+^, and CD86 expression in CT26 tumors.

### 3.5. Simultaneous Combination Therapy Enhanced CD8^+^ PD-1^+^ T-Cell Infiltration in the TME

**Figure 5:**
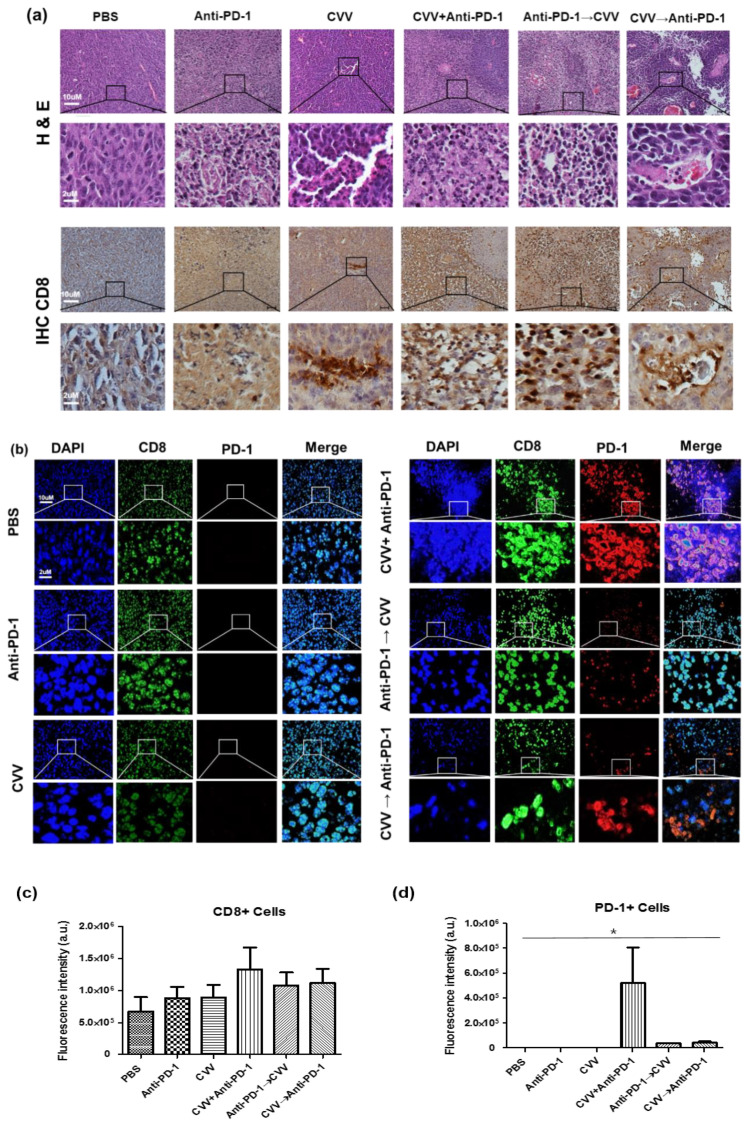
Simultaneous combination therapy enhanced CD8^+^ PD-1^+^ T-cell infiltration in the tumor microenvironment (TME). After 40 days, the mice were euthanized and tumors collected for hematoxylin and eosin (H&E) staining, immunohistochemistry (IHC), and immunofluorescence analysis. (**a**) Representative images of hematoxylin & eosin (H&E) staining and immunohistochemistry (IHC). (**b**) Immunofluorescence of CT26 tumors. (**c**) Fluorescence intensity of CD8^+^ cells. (**d**) Fluorescence intensity of PD-1^+^ cells. * *p* < 0.05, one-way analysis of variance (ANOVA).

To evaluate immune cell infiltration in the TME, CT26 tumors were harvested and the infiltration of CD8^+^ PD-1^+^ T-cells was examined. The histological analysis showed enhanced tumor cell destruction in combination therapy groups. The immunohistochemistry (IHC) analysis of CT26 tumors was correlated with enhanced infiltration of CD8^+^ T-cells in the CVV+anti-PD-1 group (Figure 5a). Immunofluorescence staining of CT26 tumors indicated enhanced infiltration of CD8^+^ PD-1^+^ T-cells in the CVV+anti-PD-1 group (Figure 5b–d). In Figure 5b, green fluorescence indicates CD8^+^ cells with red fluorescence as PD-1^+^ cells. These colors indicate PD-1 expression was observed, along with CD8 expression (CD8^+^PD-1^+^cells) in CVV+anti-PD-1 received group. Weekly CVV+anti-PD-1 combination therapy upregulated CD8^+^ and PD-1^+^ T-cells and tumor suppression in CT26 tumor-bearing mice. Our results differed from those of other studies [27,28,29] that showed the impact of PD-L1 expression on the TME. In our study, interestingly, CD8^+^ PD-1^+^ T-cell infiltration was more likely due to the simultaneous activation of both arms of the antitumor immune system. In this case, CD8^+^ PD-1^+^ T-cell infiltration in the TME might lead to tumor suppression and regression.

### 3.6. PD-1 and PD-L1 Expression in the TME and Its Correlation with Therapeutic Efficacy

**Figure 6:**
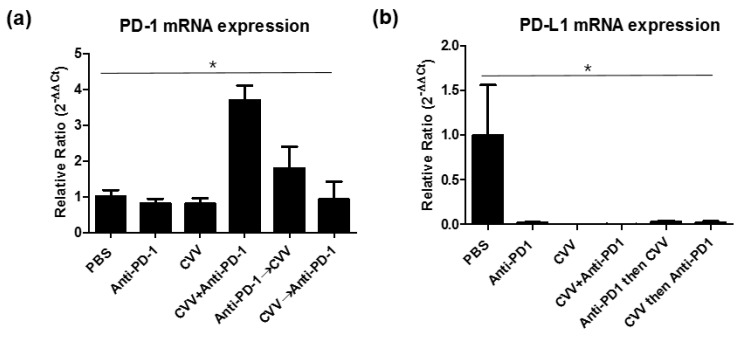
mRNA quantification of PD-1, and PD-L1 from CT26 tumors. After 40 days, tumors derived from responders were harvested, total RNA was extracted, and cDNA was synthesized for mRNA quantification. (**a**) PD-1 expression. (**b**) PD-L1 expression from different groups. The relative ratio (β-actin used for normalization) was used to quantify mRNA expression. mRNA, messenger RNA; PD-1, programmed cell death protein-1. * *p* < 0.05, one-way ANOVA.

Messenger RNA (mRNA) expressions were quantified to confirm the immune-active TME. RNA was extracted from harvested tumors, and complementary DNA (cDNA) was synthesized from the extracted RNA. CD8a, PD-1 and PD-L1 mRNA expressions were quantified from the cDNA. PD-1 mRNA expression analysis indicated the highest PD-1 upregulation in the CVV+anti-PD-1 group (Figure 6a), while PD-L1 mRNA expression was almost minimal in all treatment groups (Figure 6b). Some studies have reported that PD-L1 upregulation in tumors is associated with enhanced therapeutic efficacy [27,30], while other studies have reported that PD-L1 expression is not associated with treatment efficacy [31,32]. We found that CVV+anti-PD-1 combination therapy upregulates PD-1 expression in the TME. PD-L1 expression was downregulated in all the treatment groups. PD-1 was expressed by tumor-infiltrated lymphocytes (TILs), TAMs, and other immune cells in the TME. Therefore, PD-1 upregulation was more likely to be associated with the slow tumor burden in the CVV+anti-PD-1 group.

### 3.7. M2 and M1 Polarization of TAMs in the TME

**Figure 7:**
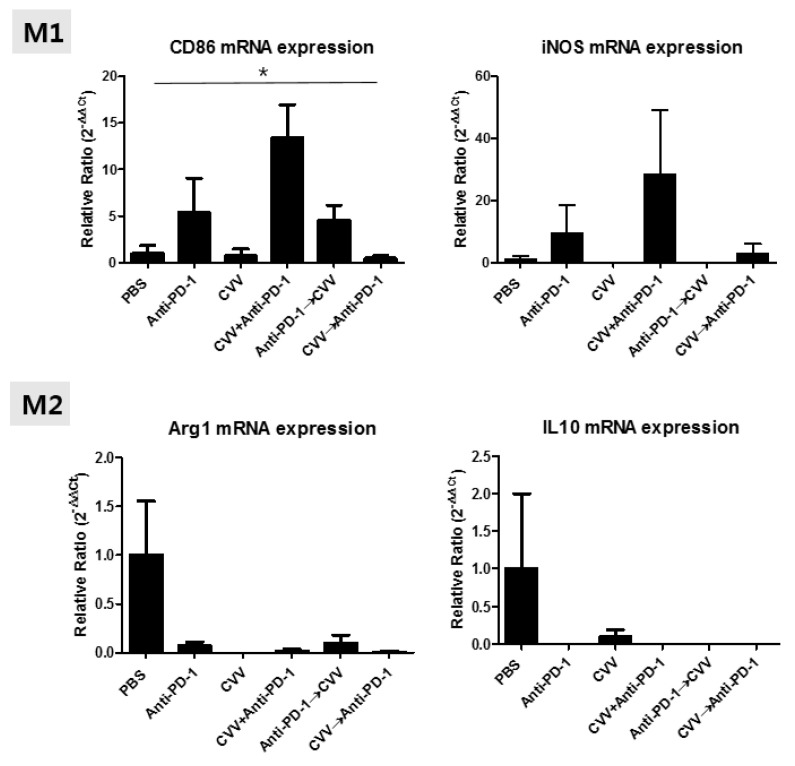
mRNA expression patterns of M1 (CD86 and iNOS) and M2 (Arg1 and IL10) markers from CT26 tumors. After 40 days, tumors derived from responders were harvested, total RNA was extracted, and cDNA was synthesized for mRNA quantification.* *p* < 0.05, one-way ANOVA.

To check the TAM status in the TME, we evaluated mRNA expressions of M1 (CD86 and inducible nitric oxide synthase [iNOS]) and M2 (Arg1 and IL-10) macrophage markers. We found expression patterns for them in CT26 tumors, indicating minimal Arg1 and IL10 expression in all groups (Figure 7). This minimal expression might be associated with the low number of M2 macrophages in the TME. In the case of the M1 marker, CD86 and iNOS expressions were upregulated in the CVV+anti-PD-1 group (Figure 7). We observed low CD86 expression in the CVV and CVV→anti-PD-1 groups, while the anti-PD-1 and anti-PD-1→CVV groups showed almost the same level of CD86 expression. This may be because of continuous viral injection. Therefore, mono- and sequential therapy showed low M1 macrophage polarization, indicating the importance of combination therapy. Dendritic cells also express CD86. CD86 provides, along with CD80, costimulatory signals important for T cell activation and survival [33,34]. Therefore, CD86 upregulation maybe correlated with antitumor immunity and T cell activation.

### 3.8. Activation of the Central Immune System by Simultaneous Combination Therapy

**Figure 8:**
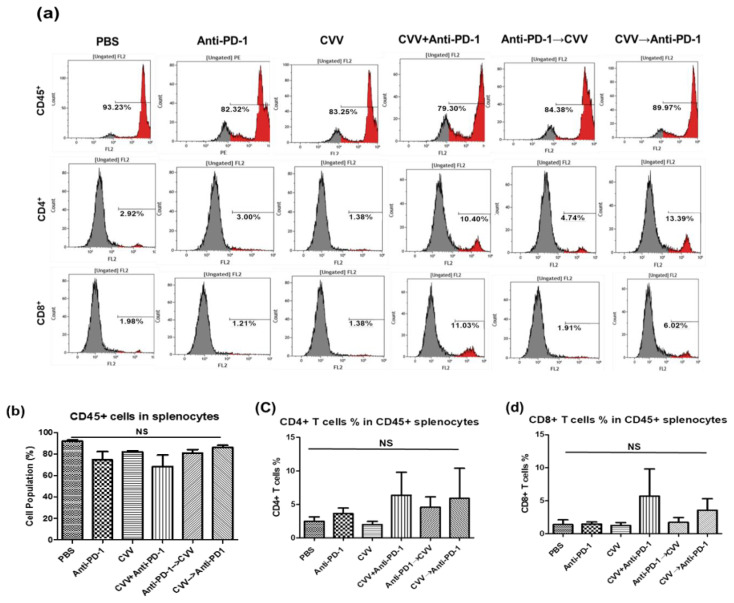
The central immune system is activated by simultaneous combination therapy. Splenocytes enumeration of CT26 tumor–bearing Balb/c mice. After 40 days, spleens were harvested from responders and a single-cell preparation was made for flow cytometry. CD45^+^, CD8^+^, and CD4^+^ cell populations were quantified. (**a**) Representative images of CD45^+^, CD4^+^, and CD8^+^ T-cell histograms in all six groups.(**b**) Total number of CD45^+^ cells in splenocytes.(**c**) CD4^+^ T-cells % in CD45^+^ cells in all six groups. (**d**) CD8^+^ T-cells % in CD45^+^ cells in all six groups.

To evaluate the central immune system, we harvested the mice spleens and made a single-cell preparation with red blood cell (RBC) lysis buffer. After cell counting, 10,000 cells were stained with mouse anti-CD45, anti-CD8, or anti-CD4 antibodies for immunophenotyping. Flow cytometry of splenocyte immunophenotyping showed upregulation of CD8^+^ and CD4^+^ T-cells in the CVV+anti-PD-1 group (Figure 8). CD4^+^ T-cells levels were almost the same in monotherapy groups, while combination therapy groups showed CD4^+^ T-cell upregulation. The CVV→anti-PD-1 group showed slight CD8^+^ T-cell upregulation, while the CD8^+^ T-cell levels were the same in the other groups. These results indicate the importance of CVV in combination therapy. Our results indicated that the activation of the central immune system is also required for the therapeutic efficacy of combination therapy.

## 4. Discussion

Although most OVT-based studies indicate enhanced therapeutic efficacy in various cancer models, the overall survival rate and objective response are limited to specific patient types [35]. OVT is a type of immunotherapy that boosts innate and adaptive immunity against cancer. The OVT therapeutic efficacy might vary among individuals due to the intrinsic clearance ability of OVs by the immune system. Time interval, dosage, and route of administration play an important role in treatment outcomes [22]. Such varied therapeutic responses among individual mice has been reported in OV and ICB-based combination studies [36] and in a recently conducted clinical study, which used combined talimogenelaherparepvec and pembrolizumab therapy to treat advanced melanoma patients. The phase 1b clinical trial showed an overall response rate of 62% and a complete response rate of 33% in the patients [37]. All of these results indicate variations in immune cell infiltration in the TME. The therapeutic efficacy of ICB has also been extensively studied at both preclinical and clinical levels [38,39]. Although complete regression and survival rate were significantly increased in these studies, the immunosuppressive nature and stromal cells of the TME restrict tumor regression. In the TME, various cells also express PD-1 on their surfaces and affect the therapeutic efficacy of immunotherapies. The roles of PD-1^+^ TAMs are well explained in the CT26 tumor model. Their expression is negatively correlated with the phagocytic ability of TAMs, and treatment with PD-1 block enhances this phagocytic ability [40].

In this study, we used weekly CVV and/or anti-PD-1 monotherapy and combination therapy in order to determine the individual impact of CVV and anti-PD-1 on the TME. We also analyzed the effects of concurrent and sequential CVV and anti-PD-1 injections. This approach reveals the roles of time interval and dosage in combination therapy. Our results showed that the upregulation of CD8^+^ and PD-1^+^ T-cell infiltration is positively correlated with the responders, with significant tumor regression and survival in the CVV+anti-PD-1 group. Expansion of CD8^+^ and PD-1^+^ T-cells is reportedly associated with enhanced therapeutic efficacy of adoptive T-cell therapy in melanoma and adenocarcinoma [41]. PD-1 expression on TILs is positively correlated with CD8^+^ tumor-reactive repertoire infiltrating human melanoma tumors [42]. Our results support the robust activation of CD8^+^ and PD-1^+^ T-cells in the TME in the CVV+anti-PD-1 group. The reason for this is that CVV infection probably increases TAA generation from tumor cells, and subsequently, these TAAs are captured by antigen-presenting cells and activate CD8^+^ T-cells. Activated CD8^+^ T-cells possibly express the PD-1 receptor on their surface.

We also observed a delayed tumor burden in other combination therapy groups, possibly because of the delayed activation of CD8^+^ T-cells in the TME. CD8^+^ T-cell infiltration in the TME depends on activation of the central immune system [43]. A lack of this systemic activation and low CD8^+^ T-cell infiltration in the TME could be associated with nonresponders. Therefore, the systemic immune activity response to CVV and anti-PD-1 treatment should be analyzed. CVV infection might accelerate central immunity through adaptive immunity. The importance of central immunity in the antitumor immunity response was recently demonstrated in a mouse model [43]. We analyzed the status of central immune activity using immunophenotyping of splenocytes (CD45^+^, CD8^+^, and CD4^+^ cell counting) and observed similar levels of CD45^+^ cells in all groups, suggesting immune activated statuses against tumor cells. Interestingly, both CD8^+^ and CD4^+^ cells were upregulated in the CVV+anti-PD-1 group. The upregulated CD8^+^ T-cells here might be tumor cell specific, because the CVV group showed decreased CD8^+^ T-cell proliferation. These results may indicate the stimulation of systemic immune activity against CT26 tumors by weekly simultaneous CVV+anti-PD-1 combination therapy.

The PD-L1 and PD-1 axis plays an important role in the therapeutic efficacy of immunotherapies [29,44]. Upregulated PD-L1 expression in tumors is associated with enhanced therapeutic efficacy [27,28,45]. However, discordant results of PD-L1 and PD-1 expression have been reported in some studies [31,46]. PD-L1 upregulation is linked with the inflammatory nature of the TME. In this study, we observed minimal PD-L1 expression in all treatment groups. CD8^+^ PD-1^+^ upregulation is correlated with active T-cell signaling and tumor regression [42,47]. We observed enhanced CD8^+^ PD-1^+^ T-cell infiltration in the TME in the CVV+anti-PD-1 group, which confirmed the antitumor immune activity mechanism of combination therapy. Recent studies have explored the importance of PD-1 expression on TAMs, and their roles have been revealed in various cancer models [39,40]. Macrophage polarizations determine the therapeutic efficacy of TAMs in the TME. There are two types of TAMs reported in the TME on the basis of their roles in tumor progression. M1 phenotypic TAMs are associated with tumor suppression, while and M2 phenotypic TAMs are closely linked with tumor progression and poor prognosis [48]. We observed CD86 (M1 macrophage marker) mRNA upregulation in the CVV+anti-PD-1 and anti-PD-1→CVV groups, which may indicate M1 macrophage polarization by CVV+anti-PD-1 combination therapy.

The enhanced therapeutic efficacy of immunotherapies depends on various co-stimulatory and inhibitory receptors of immune cells [49]. CD28, the CD8^+^ T-cell co-stimulatory receptor, plays a vital role in PD-1-mediated inhibition. PD-1 suppresses CD8^+^ T-cell function predominantly by inactivating CD28 signaling [50]. CD28 expression reinvigorates CD8^+^ CD28^+^ PD-1^+^ T-cells. Anti-PD-1 therapy in lung cancer patients shows upregulation of CD8^+^ CD28^+^ PD-1^+^ T-cells in blood [51]. However, we did not check for CD28 expression; instead, we checked for CD86, the ligand for CD28 expression, as an M1 macrophage marker. CD86mRNA upregulation in groups treated at different time points may reflect the effect of combination therapy on co-stimulatory receptors.

**Figure 9:**
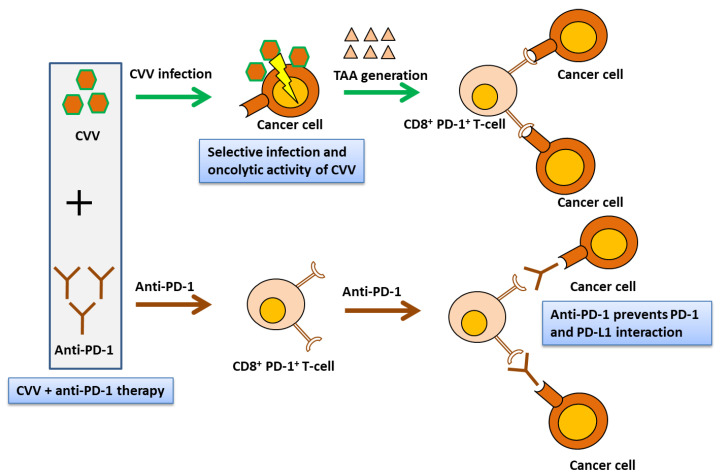
CVV and anti-PD-1-based combination therapy restores antitumor immunity in the TME. Simultaneous CVV and anti-PD-1 combination therapy induced CD8^+^ PD-1^+^ T-cell infiltration in the TME. Oncolysis of cancer cells by CVV might activate cancer-specific CD8^+^ PD-1^+^ T-cells. Oncolysis and decrease of PD-1 and tumor cell interaction by anti-PD-1 might inhibit tumor growth. CVV, cancer-favoring oncolytic vaccinia virus; PD-1, programmed cell death protein-1; TME, tumor microenvironment; TAA, tumor-associated antigen.

Therapeutic transgene-tailored OVs lead to significant tumor regression in various cancer models [3,17,52,53]. In this study, we used CVV, which was constructed by replacing the vTK gene from a naturally evolved cancer-favoring Wyeth strain vaccinia virus strain, and then it was isolated and characterized by repeated replication from tumor tissue lysis [23]. CVV was injected intratumorally because a systemically administrated virus is cleared by reticuloendothelial phagocytes in the spleen and liver. We confirmed the therapeutic efficacy of CVV+anti-PD-1 combination therapy against subcutaneous CT26 tumors in a syngeneic mouse model. This therapeutic efficacy was mediated by the infiltration of CD8^+^ PD-1^+^ T-cells, which may directly have correlated with immune modulation of the TME (Figure 9). Our results indicated immune modulation in the TME using combination immunotherapy and strongly suggested feasibility of VV and ICB-based combination therapy in preclinical models. This study has potential limitation due to the use of a subcutaneous tumor model to reflect the complex tumor and immune microenvironment. However, immune cell infiltration in TME was nearly correlated to orthotopic models [54,55]. In order to explore more insightful mechanisms underlying therapeutic efficacy and statuses of other immune checkpoint molecules, more studies with syngeneic orthotopic models are required.

## 5. Conclusions

The weekly combined immunotherapy of locally administrated CVV and systemically administrated anti-PD-1 synergistically induces CD8^+^ PD-1^+^ T-cell infiltration in the TME, which might be associated with tumor regression in a syngeneic immunocompetent mouse CRC model. In particular, the concurrent administration of CVV and antiPD-1, rather than scheduled repeated therapy, actively induces CD8^+^ PD-1^+^ T-cell infiltration in the TME. Increased CD8^+^ and CD4^+^ T-cell populations in splenocytes support the activation of the central immune system by CVV+anti-PD-1 combination therapy.

## Figures and Tables

**Table 1:** Primers used in this study.

Name *	Sequence (5′–3′)
Mouse CD8 Forward (mCD8a_F)	CAGAGACCAGAAGATTGTCG
Mouse CD8 Reverse (mCD8a_R)	TGATCAAGGACAGCAGAAGG
Mouse PD-1 Forward (mPD-1_F)	CACAGTGTCAGAGGGAGCAA
Mouse PD-1 Reverse (mPD-1_R)	TTGGGCAGCTGTATGATCTG
Mouse PD-L1 Forward (mPD-L1-F)	CGAATCACGCTGAAAGTCAA
Mouse PD-L1 Reverse (mPD-L1-R)	GCTGGTCACATTGAGAAGCA
Mouse CD86 Forward (mCD86-F)	GCCCATTTACAAAGGCTCAA
Mouse CD86 Reverse (mCD86-R)	TGTTCCTGTCAAAGCTCGTG
Mouse iNOS Forward (miNOS-F)	CTCACTGGGACAGCACAGAA
Mouse iNOS Reverse (miNOS-R)	GGTCAAACTCTTGGGGTTCA
Mouse Arg1 Forward (mArg1-F)	CGCCTTTCTCAAAAGGACAG
Mouse Arg1 Reverse (mArg1-R)	ACAGACCGTGGGTTCTTCAC
Mouse IL-10 Forward (mIL-10-F)	GCCTTATCGGAAATGATCCA
Mouse IL-10 Reverse (mIL-10-R)	TTTTCACAGGGGAGAAATCG
Mouse β-Actin Forward (BAT-Fw)	GTCCCTCACCCTCCCAAAAG
Mouse β-Actin Reverse (BAT-Re)	GCTGCCTCAACACCTCAACCC

Note: * CD8, cluster of differentiation 8; PD-1, programmed cell death-1; PD-L1, programmed cell death -ligand 1; CD86, cluster of differentiation 86; iNOS, inducible nitric oxide synthase; Arg1, arginase 1; IL-10, interleukin 10.

## Supplementary Materials

The following are available online at https://www.mdpi.com/2076-393X/8/2/321/s1, Figure S1: Survival curve analyses.
