# Supplementary material for: Overcoming Tumor Resistance to Oncolyticvaccinia Virus with Anti-PD-1-Based Combination Therapy by Inducing Antitumor Immunity in the Tumor Microenvironment
Source: Vaccines (Basel). 2020 Jun 19;8(2):321. doi: 10.3390/vaccines8020321 (PMC7350271; doi:10.3390/vaccines8020321)
Supplement: Supplementary file 1 [file vaccines-08-00321-s001.pdf]

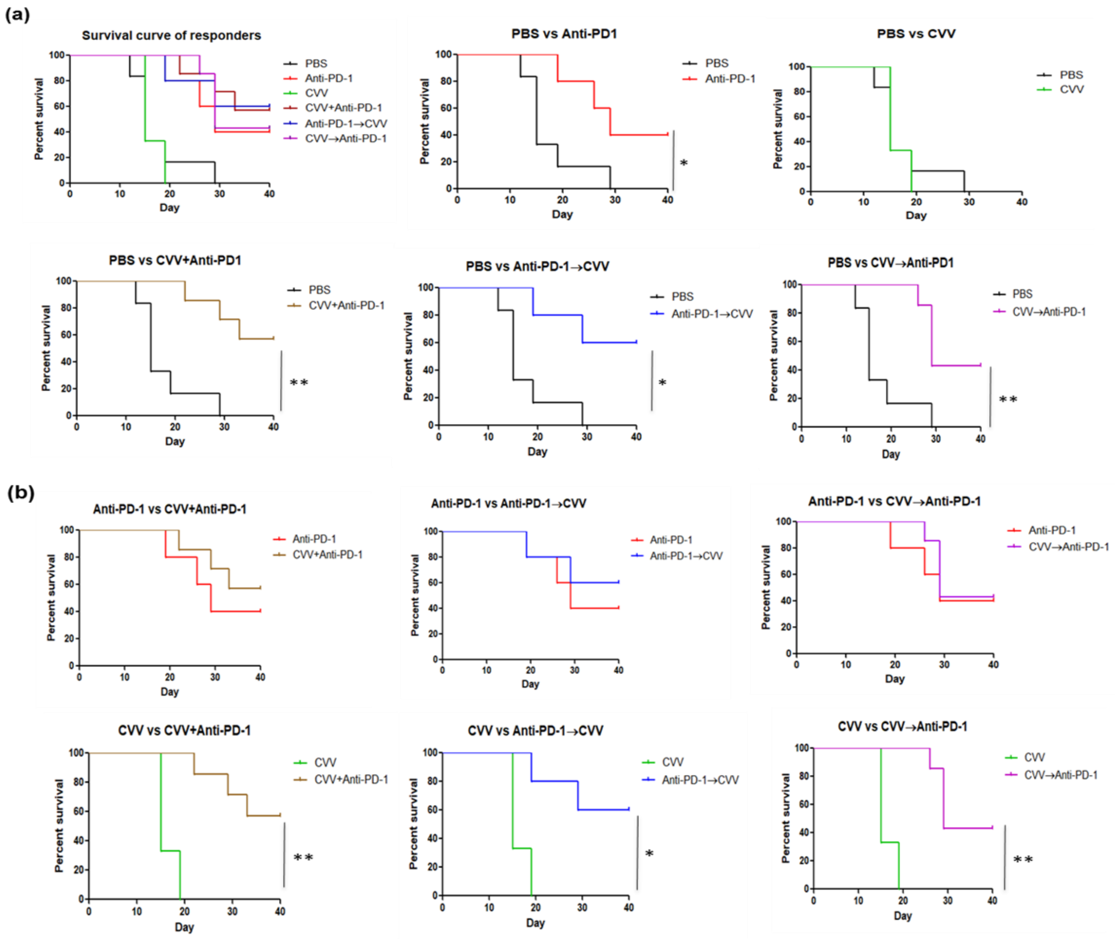

Figure S1. Survival curve analyses. Kaplan–Meier curves were plotted on the basis of tumor volume. Tumor volume >1000 mm^3 was considered death. (**a**) Survival curve among responders. (**b**) Survival curve among responders: monotherapy vs. combination therapy. * $p < 0.05$; ** $p < 0.01$.
